# Quality of life in unaccompanied young refugees: the role of traumatic events, post-migration stressors and mental distress

**DOI:** 10.1186/s12888-025-06975-1

**Published:** 2025-05-26

**Authors:** Maike Garbade, Selina Kappler, Jenny Eglinsky, Heinz Kindler, Rita Rosner, Cedric Sachser, Elisa Pfeiffer

**Affiliations:** 1 Department of Child and Adolescent Psychiatry/Psychotherapy, Ulm University, Ulm, Germany; 2 German Center for Mental Health (DZPG), partner site Ulm, Ulm, Germany; 3 Department of Clinical Psychology and Child and Adolescent Psychotherapy, Catholic University Eichstätt-Ingolstadt, Ingolstadt, Germany; 4 German Youth Institute, Munich, Germany; 5 Department of Clinical Child and Adolescent Psychology, Otto Friedrich University Bamberg, Bamberg, Germany; 6 Department of Clinical and Biological Psychology, Catholic University Eichstätt-Ingolstadt, Eichstätt, Germany

**Keywords:** Quality of life, Mental distress, Post-migration stressors, Post-traumatic stress disorder, Daily stressors, Unaccompanied young refugees

## Abstract

**Background:**

Unaccompanied young refugees (UYRs) are exposed to numerous potentially traumatizing events and post-migration stressors before, during, and after migration. These adverse experiences may affect their mental health and their quality of life. Consequently, this study aimed to focus on the quality of life of UYRs and identify factors that may influence it.

**Methods:**

Potentially traumatic events (PTEs) and posttraumatic stress symptoms (CATS-2), depressive symptoms (PHQ-9), anxiety symptoms (GAD-7), as well as post-migration stressors (DSSYR) and quality of life (WHOQOL-BREF) were assessed in *N* = 158 UYRs (*M*_Age_ = 16.92, *SD*_Age_ = 1.41, 84.2% male) residing in different child welfare facilities in Germany. Serial mediation models were used to examine the influence of the above-mentioned factors on quality of life.

**Results:**

UYRs reported a mean of 6.18 (*SD* = 3.27) PTEs and 9.72 (*SD* = 4.49) post-migration stressors. The average quality of life ranged between *M* = 57.72 (*SD* = 16.46) for environmental quality of life to *M* = 66.39 (*SD* = 20.71) for social quality of life. PTEs significantly reduced the reported physical (*b* = -1.78, *p* <.001), psychological (*b* = -1.15, *p* =.025), and social quality of life (*b* = -1.98, *p* <.001) of UYRs. However, these direct effects were mediated by post-migration stressors alone and in series with mental distress.

**Conclusions:**

The quality of life of UYRs was not only associated with traumatic experiences but also with post-migration stressors and mental distress. Quality of life captures additional aspects of well-being and therefore, psychosocial care should not only address mental distress but also consider quality of life to achieve a sustainable improvement in the well-being of UYRs. Changes at the political-structural level, aiming to reduce post-migration stressors, could potentially improve the quality of life of UYRs.

**Trial registration:**

German Clinical Trials Register DRKS00017453. Registered on December 11, 2019.

**Supplementary Information:**

The online version contains supplementary material available at 10.1186/s12888-025-06975-1.

## Introduction

Forced migration due to ongoing war and conflict is increasing worldwide. In 2022, the UNHCR estimated that about 40% of all displaced persons were minors, many of them unaccompanied [1]. It is widely recognized that unaccompanied young refugees (UYRs) are a highly vulnerable population, not only due to their experience of aversive events before, during, and after migration [2]. Nevertheless, it is important to keep in mind that UYRs do not necessarily develop mental disorders [3]. It is, therefore, necessary to closely examine not only the psychopathology of UYRs but also their perceived quality of life (QoL). The World Health Organization (WHO) defines quality of life as “an individual’s perception of their position in life in the context of the culture and value systems in which they live and in relation to their goals, expectations, standards, and concerns” [4]. According to this definition, QoL is a broad concept that encompasses not only the subjective assessment of one’s own mental and physical health but also that of one’s own social relationships and environmental situations. Furthermore, in the case of refugees, QoL is also linked to their successful integration into the receiving society [5] and could be a protective factor against concurrent and future stress [6].

Previous studies with diverse refugee populations, such as accompanied minors or adult refugees, have reported lower levels of QoL compared to non-refugee youth [7], to the general population [8, 9] or to norm data from the respective region [10–12]. Studying different dimensions of QoL reveals differences in levels, highlighting the complexity of the construct. For instance, a systematic review by Gagliardi et al. [13] shows higher scores in environmental QoL and lower scores in psychological QoL among adult refugees but reports a wide heterogeneity among the included studies. Furthermore, factors might influence the dimensions of QoL differently [13]. Compared to adult refugees, UYRs face different life realities, for example regarding accommodation or social challenges. Thus, the results of studies with adult refugees are not transferable to UYRs.

Nevertheless, studies have mostly focused on adult refugees and evidence for refugee youth is scarce. This is surprising because it is crucial to identify the factors that might contribute to the QoL of refugee youth in order to strengthen their developmental stage and provide adequate support for their future life [10, 14].

Previous research has discussed possible factors that might have impact QoL in refugees. First, in a systematic review of studies with refugees, it was found that the experience of potentially traumatic events (PTEs) can impact perceived QoL in refugees [13]. Second, due to their stressful experiences before, during, and after migration, refugees often report mental health problems such as posttraumatic stress disorder, depression, or anxiety [3], which are associated with QoL in refugees [5]. Third, also after resettlement in the host society, refugees are confronted with daily postmigration stressors such as discrimination, uncertainties related to their asylum status, or failure to meet their basic needs [15], all of which have been shown to be associated with lower perceived QoL [8].

According to the *ecological model of refugee distress* [16], which highlights the importance of both PTEs and post-migration stressors on mental health, previous research has shown that postmigration stressors had an additional impact on mental distress among UYRs, beyond the impact of PTEs [17]. Similarly, but for the context of QoL, it has been shown that post-migration stressors might have a stronger impact on QoL than PTEs [18, 19]. Studies investigating the association between PTEs and QoL have reported mediating roles of mental distress [20, 21] and post-migration stressors [18]. To our knowledge, one of the first studies to investigate the interaction between PTEs, post-migration stressors, mental distress, and QoL in refugee youth was conducted by Dangmann et al. [19]. For *N* = 160 Syrian refugee youth recently resettled in Norway, they reported that higher levels of PTEs were associated with reduced QoL. However, this relation was mediated by post-migration stressors alone and in series with mental distress. Despite the major relevance and novel nature of this study, some limitations restricted the generalization of the results to the overall group of young refugees. First, only Syrian refugees were included in the study. Second, due to varying asylum laws, the associated prospects of staying on and their impact on the QoL of refugees [13], there are probably differences between Norway and other host countries. Third, Dangmann et al. [19] mainly included accompanied refugee youth in their study. This could have influenced their findings because previous research has reported lower scores of QoL in UYRs compared to those who were accompanied by family members [10]. Fourth, the measures used were originally developed for adult refugees (e.g., Post-migratory Stress Scale [22]), are rather outdated (e.g., CRIES-8 [23]), or did not sufficiently differentiate the dimension of QoL as they used an overall score of QoL only, although domain-specific differences in QoL had been shown in previous studies [13].

Thus, the current study aimed to replicate and build on the findings of Dangmann et al. [19] in the context of UYRs residing in child and youth welfare (CYWS) facilities in Germany. We aimed to examine QoL by differentiating for physical, psychological, social, and environmental QoL for this specific population and by analyzing the role of PTEs, post-migration stressors, and the three most frequently reported trauma-related distresses, namely post-traumatic stress symptoms (PTSS), depression, and anxiety.

**Fig. 1:**
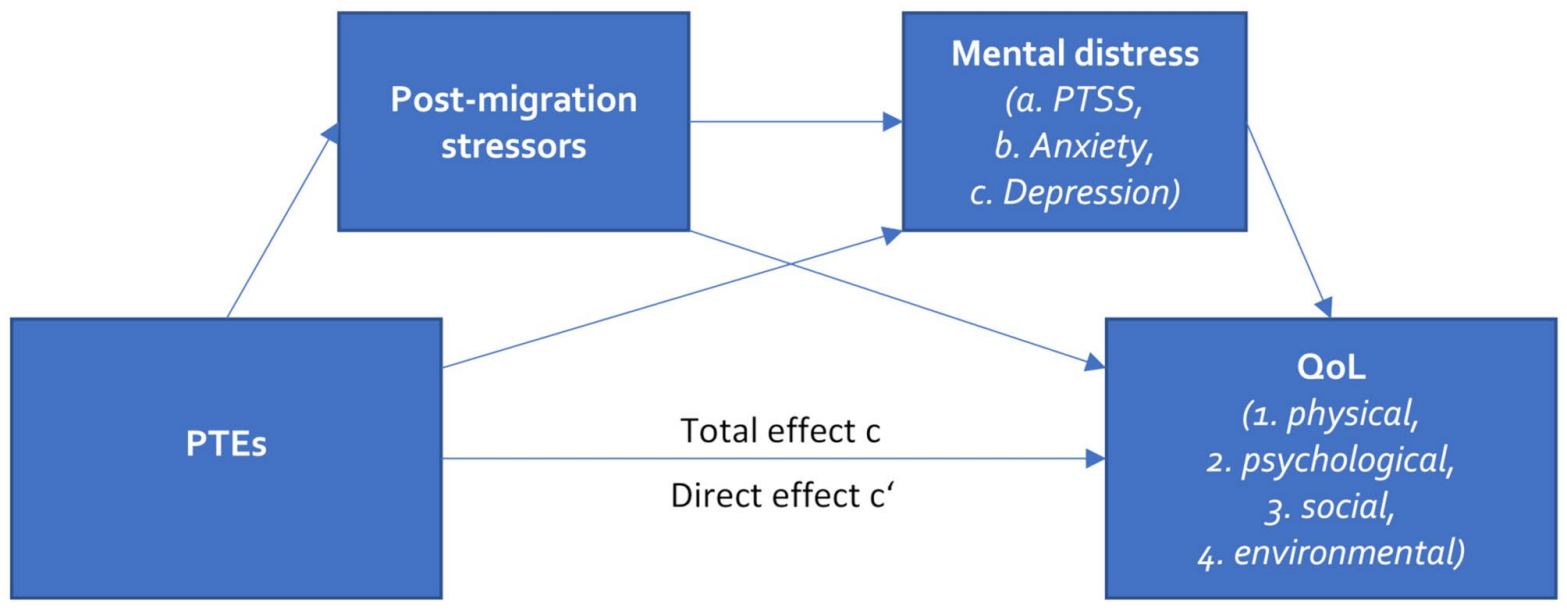
Conceptual serial mediation model Note: Adapted from Dangmann et al. [19]. PTEs = Potentially traumatic events; PTSS = Posttraumatic Stress Symptoms, QoL = Quality of Life

As depicted in Fig. 1, we expected PTEs to negatively influence the four different dimensions of QoL, and post-migration stressors and mental distress to mediate the effect of PTEs on QoL individually and in sequence.

Our hypotheses were as follows: (1) Due to the significant challenges that UYRs face even in the host context, we generally expect low reported QoL with varying levels across the examined dimensions. Furthermore, we expect that (2) PTEs negatively impact the QoL of UYRs; however, this relationship is mediated by (3) post-migration stressors alone, (4) mental distress (PTSS, depressive symptoms or severity of anxiety) alone and (5) post-migration stressors and mental distress in series.

## Methods

### Design and procedure

The present investigation constituted a secondary analysis of a subsample of the randomized controlled trial BETTER CARE (German Clinical Trials Register DRKS00017453. Registered on 11 December 2019; [24]. The project was approved by the ethics committees at Ulm University (No. 243/19) and at the Catholic University of Eichstätt-Ingolstadt (No. 004–19). Detailed information about the study design can be found elsewhere [24]. As part of the BETTER CARE trial, the present subsample was assessed using additional questionnaires about post-migration factors, such as sociocultural adaptation, social support, and post-migration stressors. For the current analysis, only baseline was used.

Inclusion criteria for participants were (1) age 12–20 years, (2) arrived in Germany as unaccompanied minors, (3) applied for asylum or intend to do so, (4) living in CYWS facility, (5) written informed consent giving by participant and legal guardian (if < 16 years at screening), and (6) reported at least one traumatic event in line with the DSM-5 A criterion. Possible participants were invited by CYWS staff to the screening appointments.

The assessment took place between July 2020 and July 2023 either in person in CYWS facilities in six German federal states or in digital form. Screening sessions were conducted in groups, with participants completing the questionnaires themselves in their native language. Trained staff and interpreters at the study sites assisted the participants to complete the measures. As compensation for their participation, participants were given vouchers worth up to €35 to be used in stores of their choice.

### Sample

Originally, *n* = 130 UYRs were planned to be included for the subsample of the BETTER CARE project. However, on top of the planned subsample, *n* = 28 UYRs voluntarily completed the additional questionnaires at baseline, resulting in a total sample of *N* = 158 UYRs, living in 35 different CYWS facilities in Germany. Of the participants, *n* = 23 (14.6%) identified themselves as female, *n* = 133 (84.2%) as male and *n* = 2 (1.3%) as diverse. The age at assessment ranged between 13 and 20 years (*M* = 16.92; *SD* = 1.41). The participants were born in 30 different countries, with most participants (*n* = 53, 33.5%) born in Afghanistan, Syria (*n* = 21, 13.3%) or Somalia (*n* = 16, 10.1%) (for a detailed description of the countries of origin, please see Table A in the supplementary material). The duration of residence in Germany at the time of assessment ranged from 0 to 90 months (*M* = 23.27, SD = 20.42). Further demographic information on the participants is presented in Table 1.

**Table 1:** Sociodemographic characteristics and means of study variables

	M (SD) or *n* (%)
Age	16.92 (1.41)
Gender	
Male	133 (84.2)
Female	23 (14.6)
Diverse	2 (1.3)
Length of stay in Germany (in months)	23.27 (20.42)
Distress regarding residential status	5.59 (3.56)
Worries about deportation	5.96 (4.20)
Contact with family	2.41 (1.93)
No (0)	50 (31.6)
Yes, once per year or less (1)	11 (7.0)
Yes, variously per year (2)	9 (5.7)
Yes, at least monthly (3)	24 (15.2)
Yes, at least weakly (4)	40 (25.3)
Yes, daily (5)	24 (15.2)
Mental distress	
CATS-2 sum score	23.23 (12.13)
PHQ-9 sum score	8.46 (5.71)
GAD-7 sum score	6.69 (4.93)
Number of PTEs	6.18 (3.27)
Number of post-migration stressors	9.72 (4.49)
Quality of life	
WHOQoL – Physical QoL	65.14 (18.76)
WHOQoL – Psychological QoL	60.35 (19.70)
WHOQoL – Social QoL	66.39 (20.71)
WHOQoL – Environmental QoL	57.72 (16.46)

Note. Values are means (SD) or *n* (%), as appropriate; CATS-2 = Child and Adolescents Trauma Screen; PHQ-9 = Patient Health Questionnaire-9; GAD-7 = Generalized Anxiety Disorder Scale-7; PTE = Potential Traumatic Event; WHOQoL = World Health Organization Quality of Life; QoL = Quality of Life

### Measures

All the questionnaires were available in English, German, French, Arabic, Dari, Farsi, Pashto, Somali, and Tigrinya. Demographic information including age, gender, country of origin, and information concerning residential status, current living situation, and family contact was assessed via self-reports.

The *Child and Adolescents Trauma Screen* (CATS-2) [25] was used to assess PTEs and PTSS according to DSM-5 and ICD-11 criteria in children and adolescents. The event checklist includes 15 items assessing natural disasters, serious accidents, experiencing or seeing violence at home or in the community, (online) sexual abuse, (cyber-)bullying, traumatic loss, medical procedures and war, for example “*threatened*,* hit or hurt badly in my family”.* Participants could answer either “yes” or “no” to indicate whether they had experienced the presented PTE or not. The severity of PTSS was measured via 25 items and a 4-point Likert scale, ranging from 0 (*never*) to 3 (*almost always*). The items map directly onto the diagnostic criteria for PTSD in DSM-5 and ICD-11, for example “*Trying not to think about what happened. Or to not have feelings about it.”.* In the current analysis, we used the DSM-5 total score ranging from 0 to 60, with higher scores indicating greater symptom loads. The CATS-2 has been validated in an international sample of traumatized children and adolescents [25] and has been used in several previous studies with UYRs, showing sufficient to good reliabilities (Cronbach’s alpha (α) = 0.75–0.83; [26, 27]). For the current sample, α was 0.93, indicating excellent reliability.

The experience of post-migration stressors was assessed using the mean score of the *Daily Stressors Scale for Young Refugees* (DSSYR) [15]. On a 4-point Likert scale from 0 (not) to 3 (very much), the participants rated the extent to which they had experienced 19 different post-migration stressors. An example item is: “*How often did you experience not enough money?”* Additionally, there was the option to indicate “*I don’t know / I don’t want to answer*”. The DSSYR was developed on the basis of the Columbia Impairment Scale (CIS [28]), the Adolescent Complex Daily Stressors Scale (ACDSS [29]), and the author’s experiences in the field and is widely used in research with UYRs (α = 0.79–0.91; [30, 31]. Nevertheless, no validation study of this measure has been published yet. The level of perceived post-migration stressors was obtained by dichotomizing scores into experiencing each stressor (yes/no), calculating mean scores, and multiplying the mean scores with the number of items. Here the aim was also to obtain the score of participants who had missing items. In the current sample, α was 0.87, indicating good reliability. Due to missing data (more than 30% of the items in the questionnaire were not answered), the mean score of 8 cases (5.06%) could not be calulated.

The *Patient Health Questionnaire* (PHQ-9) [32, 33] was used to measure symptoms of depression. The degree of impairment over the past two weeks was measured via 9 items (e.g. “*Little interest or pleasure in doing things*”) on a 4-point Likert scale ranging from 0 (*not at all)* to 3 *(nearly every day*). The PHQ-9 has been validated in many contexts and languages [32, 34] and in the current sample, α was 0.84, indicating good reliability.

Severity of anxiety was assessed using the *General Anxiety Disorder Scale* (GAD-7; [35]). On a 4-point Likert-scale ranging from 0 (*not at all)* to 3 *(nearly every day*), 7 items (e.g. “*Feeling nervous*,* anxious or on edge”)* measured the degree of impairment over the previous two weeks. Validation studies for the GAD-7 were made for many different contexts and languages [34]. In the current sample, α was 0.87, indicating good reliability.

Quality of Life was assessed using the *World Health Organization Quality of Life*,* brief version* (WHOQOL-BREF) [36]. Four domains of QoL (physical QoL, 7 items; psychological QoL, 6 items; social QoL, 3 items; environmental QoL, 8 items) were assessed using a 5-point Likert scale, ranging from 1 (*very poor/dissatisfying*,* not at all*,* never)* to 5 *(very good/satisfying*,* an extreme amount*,* extremely*,* completely*,* always)*. The physical QoL subscale evaluates aspects such as pain, physical capacity, and the ability to perform daily activities, with high scores indicating good physical health and minimal pain. An example item for the physical QoL is “*To what extend do you feel that physical pain prevents you from doing what you need to do?”.* The psychological QoL subscale measures factors such as emotional well-being, self-esteem, and the presence of negative feelings, with high scores reflecting positive emotional well-being. An example item for the psychological QoL is “*How much do you enjoy life?”.* The social QoL subscale assesses the quality of social interactions and support systems, with higher scores denoting strong and satisfying personal relationships. An example item for the social QoL is “*How satisfied are you with the support you get from your friends?”.* The environmental QoL subscale includes aspects such as financial resources, safety, access to healthcare and social services, and opportunities for recreation. Higher scores on this subscale represent a positive living environment with ample opportunities for learning and leisure. An example item for the environmental QoL is “*To what extend do you have the opportunity for leisure activities?*”. Data on the WHOQOL-BREF were missing in eight cases (5.06%). The questionnaire has been validated with diverse international samples [37]. Domain scores of the WHOQOL-BREF were calculated according to the manual [36], by calculating the mean score and converting it into a scale from 0 to 100. Values can be interpreted as percentages with higher numbers represented better perceived QoL in the respective domain. Preliminary norm data suggests average scores of 73.5 for physical QoL, 70.6 for psychological QoL, 71.5 for social QoL and 75.1 for environmental QoL [38]. In the current sample, α ranged between 0.66 and 0.78 for the subscales of the WHOQOL-BREF, indicating acceptable reliability.

### Statistical analysis

Data analysis was conducted using IBM SPSS Statistics for Windows, Version 28.0. Descriptive statistics and correlation analysis were used to explore the variables. Due to skewed data, we conducted an additional Spearman correlation analysis. Given the high intercorrelation with the variable “worries about deportation,” only “distress regarding residential status” was included.

To examine the relationships between PTEs, post-migration stressors, mental distress and QoL, serial mediation analyses were performed using the PROCESS macro (model 6) for SPSS by Hayes [39]. For each domain of the WHOQOL-BREF (1) physical QoL, (2) psychological QoL, (3) social QoL, and (4) environmental QoL), separate analyses were performed. The independent variable in all models was PTEs, with post-migration stressors as the first mediator and PTSS, severity of anxiety or depressive symptoms as one aspect of mental distress as the second mediator. This resulted in unstandardized path coefficients for total, direct, and indirect effects for each mediator isolated and in series. The total effects asses the overall relationship between PTEs and QoL. The direct effects reflect the relationship after controlling for the mediators and the indirect effects examine the roles of post-migration stressors and mental health on QoL. In total, 12 serial mediation models were conducted. Bootstrapping with 10,000 samples was employed to compute the confidence intervals (CI). Age and gender were included as control variables in our models.

All tests were two-tailed, and an alpha level of *p* <.05 was used.

## Results

Descriptive data for all study variables are presented in Table 1.

The participants reported a high number of PTEs (*M* = 6.18, *SD* = 3.27; range 0–14). The most frequently reported experiences entailed witnessing family violence (*n* = 99, 62.7%), witnessing community violence (*n* = 97, 61.4%), and being around war (*n* = 95, 60.1%). The participants experienced a high number of post-migration stressors (*M* = 9.72, *SD =* 4.49; range 0–19). The most frequently reported post-migration stressors were worries about family members in the home country (*n* = 122, 87.8%), not enough money (*n* = 117, 78.5%), and boredom (*n* = 115, 74.2%). The mean scores for PTSS (*M* = 23.23, *SD =* 12.13), depressive symptoms (*M* = 8.46, *SD* = 5.71), and severity of anxiety (*M* = 6.69, *SD* = 4.93) were all above the subclinical cut-off. The mean scores of the domains of QoL were all above the mid-point, but below preliminary norm data [38], ranging from *M* = 57.72 (*SD* = 16.46) for environmental QoL, over *M* = 60.35 (*SD* = 19.70) for psychological QoL, and *M* = 65.14 (*SD* = 18.76) for physical QoL, to *M* = 66.39 (*SD* = 20.71) for social QoL. Table 2 presents Spearman correlation coefficients for all the main study variables. PTEs were significantly correlated with physical QoL, psychological QoL and social QoL, but not with environmental QoL. Post-migration stressors were significantly correlated with all four dimensions of QoL. PTSS, depressive symptoms and severity of anxiety correlated significantly with all four dimensions of QoL.

**Table 2:** Spearman correlations between included variables

	1	2	3	4	5	6	7	8	9	10	11	12	13
1. Age													
2. Gender	0.15												
3. Length of stay in Germany	0.37***	0.17*											
4. Distress regarding residential status	0.12	0.02	− 0.09										
5. Contact with family	− 0.03	− 0.12	0.14	− 0.12									
6. PTEs	0.10	0.07	0.12	0.33***	− 0.27***								
7. CATS-2	0.15	0.14	0.13	0.39***	− 0.31***	0.60***							
8. PHQ-9	0.11	0.16*	0.21**	0.26***	− 0.22**	0.39***	0.73***						
9. GAD-7	0.05	0.11	0.17*	0.35***	− 0.26***	0.46***	0.79***	0.78***					
10. Post-migration stressors	0.08	0.04	0.01	0.24**	− 0.07	0.25**	0.48***	0.52***	0.40***				
11. WHOQoL physical	− 0.13	− 0.13	− 0.08	− 0.18*	0.21*	− 0.29***	− 0.56***	− 0.63***	− 0.56***	− 0.47***			
12. WHOQoL psychological	0.04	− 0.06	− 0.02	− 0.13	0.14	− 0.16*	− 0.49***	− 0.61***	− 0.56***	− 0.49***	0.68***		
13. WHOQoL social	− 0.08	− 0.25**	− 0.03	− 0.16	0.20*	− 0.32***	− 0.42***	− 0.37***	− 0.38***	− 0.45***	0.53***	0.50***	
14. WHOQoL environmental	0.05	− 0.15	0.18*	− 0.14	0.05	− 0.03	− 0.22**	− 0.33***	− 0.27***	− 0.51***	0.50***	0.55***	0.34***

Note. *p <.05 **p <.01 ***p <.001; PTE = Potential Traumatic Event; CATS-2 = Child and Adolescents Trauma Screen; PHQ-9 = Patient Health Questionnaire-9; GAD-7 = Generalized Anxiety Disorder Scale-7; WHOQoL = World Health Organization Quality of Life

### Serial mediation analysis

**Fig. 2:**
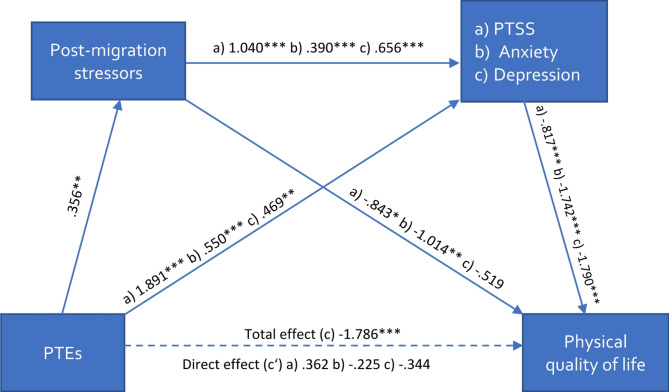
Serial multiple mediation model on physical quality of life Note: Values represent unstandardized b-values, * *p* <.05, ** *p* <.01, *** *p* <.001; (a) values for the model with PTSS, (b) values for the model with severity of anxiety, (c) values for the model with depressive symptoms; PTEs = Potentially traumatic events; PTSS = Posttraumatic Stress Symptoms

**Fig. 3:**
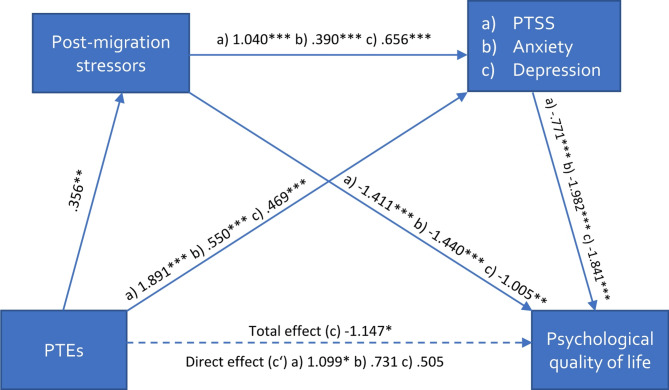
Serial multiple mediation model on psychological quality of life Note: Values represent unstandardized b-values, * *p* <.05, ** *p* <.01, *** *p* <.001; (a) values for the model with PTSS, (b) values for the model with severity of anxiety, (c) values for the model with depressive symptoms; PTEs = Potentially traumatic events; PTSS = Posttraumatic Stress Symptoms

**Fig. 4:**
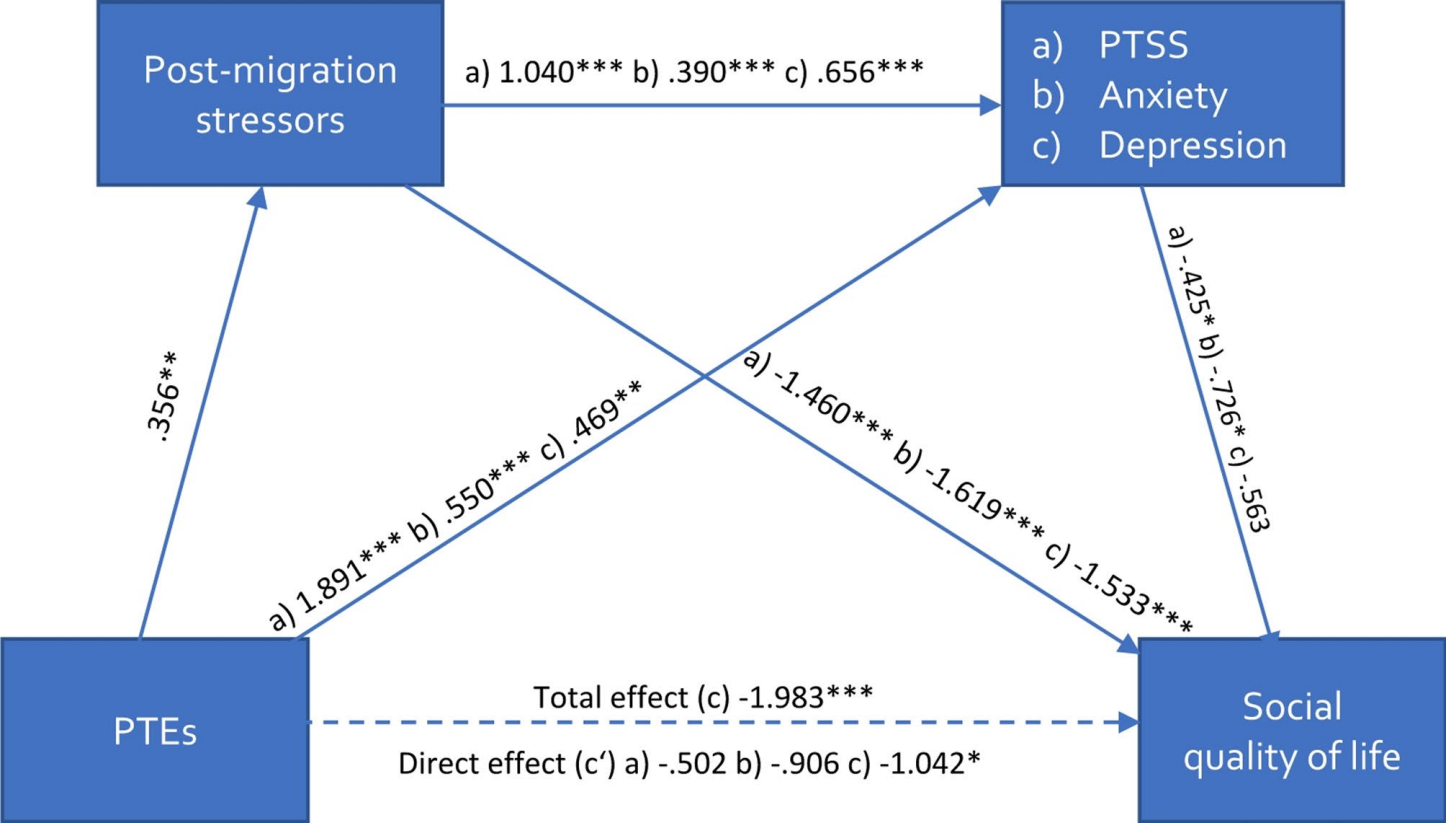
Serial multiple mediation model on social quality of life Note: Values represent unstandardized b-values, * *p* <.05, ** *p* <.01, *** *p* <.001; (a) values for the model with PTSS, (b) values for the model with severity of anxiety, (c) values for the model with depressive symptoms; PTEs = Potentially traumatic events; PTSS = Posttraumatic Stress Symptoms

**Fig. 5:**
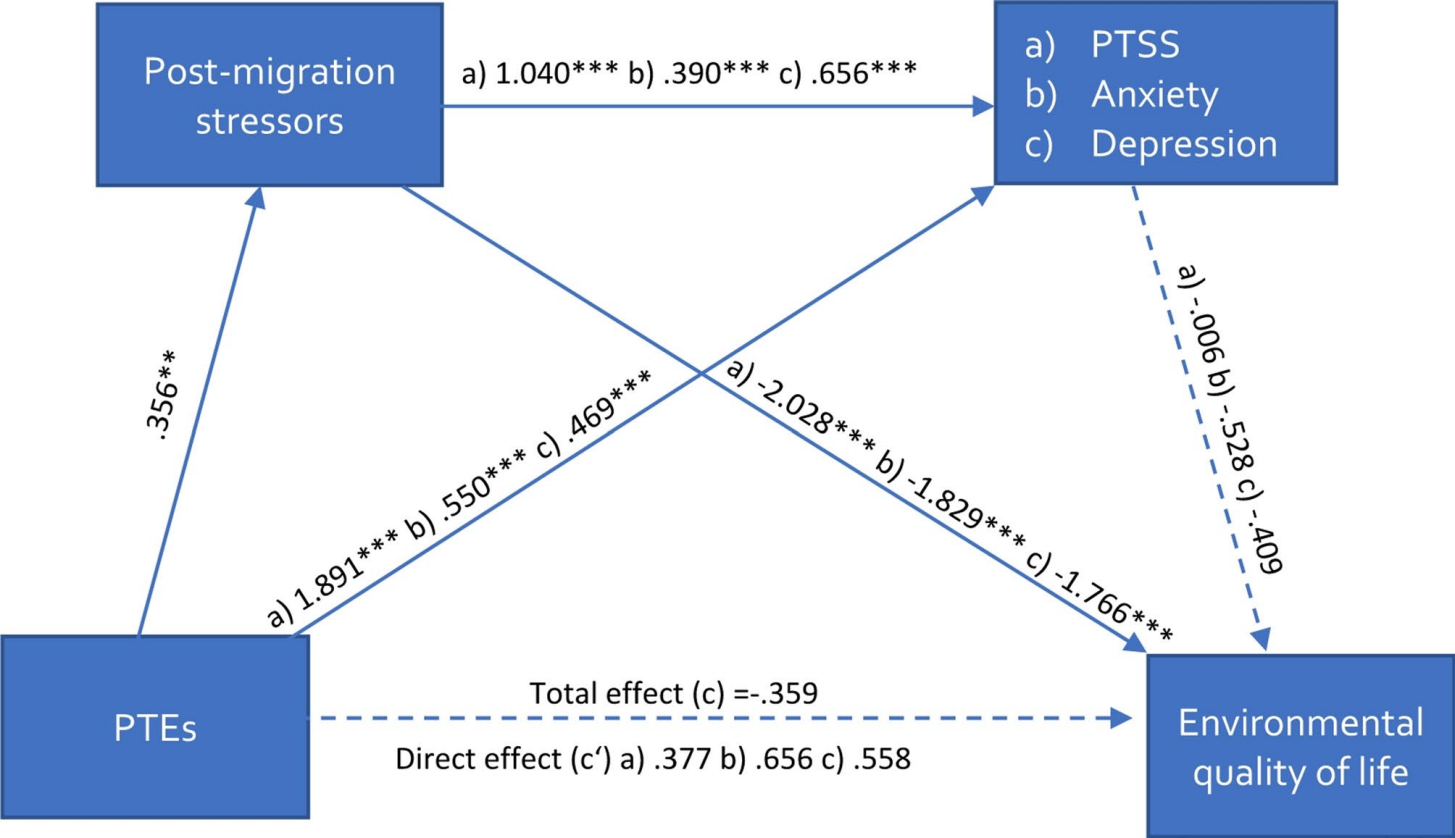
Serial multiple mediation model on environmental quality of life Note: Values represent unstandardized b-values, * *p* <.05, ** *p* <.01, *** *p* <.001; (a) values for the model with PTSS, (b) values for the model with severity of anxiety, (c) values for the model with depressive symptoms; PTEs = Potentially traumatic events; PTSS = Posttraumatic Stress Symptoms

The results of the serial mediation analysis are presented in Figs. 2, 3, 4 and 5; Table 3.

**Table 3:** Serial mediation analysis

*Outcome*	*Mental distress (M2)*	Total effect c			Direct effect c‘			Total indirect effect M1M2				Indirect effect M1				Indirect effect M2			
		*b*	SE (b)	*p*	*b*	SE (b)	*p*	*b*	SE (b)	95% CIs		*b*	SE (b)	95% CIs		*b*	SE (b)	95% CIs	
										LL	UL			LL	UL			LL	UL
Physical QoL	a. PTSS	-1.786***	0.47	< 0.001	0.36	0.48	0.449	**− 0.30**	0.14	− 0.63	− 0.07	**− 0.30**	0.17	− 0.67	− 0.03	**-1.55**	0.37	-2.34	− 0.91
	b. Anxiety				− 0.23	0.43	0.601	**− 0.24**	0.12	− 0.52	− 0.06	**− 0.36**	0.18	− 0.75	− 0.07	**− 0.96**	0.27	-1.55	− 0.50
	c. Depression				− 0.34	0.41	0.404	**− 0.42**	0.19	− 0.84	− 0.11	**− 0.19**	0.35	− 0.53	− 0.09	**0.84**	0.25	-1.38	− 0.38
Psychologi-cal QoL	a. PTSS	-1.15*	0.51	0.025	1.10	0.50	0.030	**− 0.29**	0.14	− 0.60	− 0.07	**− 0.50**	0.22	− 0.98	− 0.12	**-1.46**	0.37	-2.25	− 0.80
	b. Anxiety				0.73	0.43	0.091	**− 0.28**	0.14	− 0.61	− 0.07	**− 0.51**	0.22	− 0.97	− 0.13	**-1.09**	0.30	-1.76	− 0.57
	c. Depression				0.51	0.42	0.234	**− 0.43**	0.20	− 0.88	− 0.11	**− 0.36**	0.18	− 0.75	− 0.05	**− 0.86**	0.26	-1.40	− 0.39
Social QoL	a. PTSS	-1.98***	0.52	< 0.001	− 0.50	0.58	0.385	**− 0.16**	0.10	− 0.38	− 0.01	**− 0.52**	0.24	-1.05	− 0.12	**− 0.80**	0.37	-1.55	− 0.10
	b. Anxiety				− 0.91	0.52	0.086	− 0.10	0.08	− 0.29	0.00	**− 0.58**	0.25	-1.12	− 0.15	− 0.40	0.23	− 0.91	0.00
	c. Depression				-1.04*	0.51	0.044	− 0.13	0.11	− 0.39	0.04	**− 0.55**	0.25	-1.10	-1.3	− 0.26	0.20	− 0.69	0.08
Environ-mental QoL	a. PTSS	− 0.36	0.44	0.412	0.38	0.46	0.416	− 0.02	0.05	− 0.13	0.09	**− 0.72**	0.27	-1.27	− 0.22	− 0.01	0.26	− 0.52	0.49
	b. Anxiety				0.66	0.41	0.113	− 0.07	0.06	− 0.22	0.00	**− 0.65**	0.25	-1.16	− 0.19	− 0.29	0.17	− 0.68	0.02
	c. Depression				0.56	0.40	0.168	− 0.10	0.09	− 0.32	0.02	**− 0.63**	0.24	-1.12	− 0.19	− 0.19	0.14	− 0.47	0.07

Note. Controlled for age and gender; M1 = Mediator 1 (post-migration stressors); M2 = Mediator 2 (PTSS, depression or anxiety); * *p* <.05, ** *p* <.01, *** *p* <.001; PTSS = Post-traumatic stress symptoms; QoL = Quality of life

As expected in our hypothesis, physical QoL was significantly associated with the number of reported PTEs. This effect was indirectly mediated through the experiences of post-migration stressors and mental distress in series (*total indirect effect*: b_Post−migration stressors − PTSS_ = − 0.30, CI [-0.63; − 0.07]; b_Post−migration stressors − Depression_ = − 0.42, CI [-0.84; − 0.11]; b_Post−migration stressors − Anxiety_ = − 0.24, CI [-0.52; − 0.06]). These models accounted for 41–44% of the variance. Similarly, the effect of PTEs on psychological QoL was indirectly mediated through the experiences of post-migration stressors and mental distress in series (*total indirect effect*: b_Post−migration stressors − PTSS_ = − 0.29, CI [-0.60; − 0.07]; b_Post−migration stressors − Depression_ = − 0.43, CI [-0.88; − 0.11]; b_Post−migration stressors − Anxiety_ = − 0.28, CI [-0.61; − 0.07]). These models accounted for 40–46% of the variance. The effect of PTEs on social QoL was indirectly mediated through post-migration stressors and PTSS in series (*total indirect effect*: b_Post−migration stressors − PTSS_ = − 0.16, CI [-0.38; − 0.01]) but not for post-migration stressors and depressive symptoms or severity of anxiety in series (*total indirect effect*: b_Post−migration stressors − Depression_ = − 0.13, CI [-0.39; 0.04]; b_Post−migration stressors − Anxiety_ = − 0.10, CI [-0.29; 0.00]. These models accounted for 30–32% of the variance. However, when controlling for severity of anxiety or depressive symptoms, post-migration stressors acted as an independent mediator (b = − 0.55, CI [-1.10; − 0.13]; b = 0.58, CI [-1.12; − 0.15]. No mediation effect was found for environmental QoL, but an indirect effect was identified through the experience of post-migration stressors solely when controlling for PTSS (b = − 0.72, CI [-1.27; − 0.22], depressive symptoms (b = − 0.63, CI [-1.12; − 0.19], and severity of anxiety (b = − 0.65, CI [-1.16; − 0.19].

## Discussion

The aim of this study was to examine QoL in UYRs and how adverse experiences before, during, and after migration may impact their QoL. Similar to previous research [26, 27, 30], we have found high prevalence rates of adverse experiences in UYRs, such as PTEs, post-migration stressors and overall high mental distress. Furthermore, PTEs, post-migration stressors, mental distress and QoL in UYRs were significantly associated. Thus, these findings highlight the importance of both post-migration stressors and mental distress when aiming to improve QoL in UYRs.

### Quality of life in UYRs

The participants presented on average moderate QoL in all domains, with lower values than in preliminary norm data for all domains [38]. The reported QoL in the present study was lower than in adult refugees in Germany [40] and below the general population norm [38]. This highlights the vulnerability of UYRs and the need to enhance QoL in UYRs to ensure their successful integration into the new society. Nevertheless, the participants in the study by Georgiadou et al. [40] reported fewer PTEs and lower mental distress than the UYRs in the current sample. This may have resulted in higher QoL ratings compared with our study. Compared with clinical samples of adult refugees in Europe [40], the reported QoL was higher in the present study. This could be explained by the beneficial accommodation situation [41] and the associated support from professionals in the CYWS facilities [42]. Pedagogical professionals provide not only emotional support by addressing the UYRs concerns but also practical assistance and help in understanding the German bureaucratic system, including the health care system [43].

The highest scores of QoL were observed in the domain of social relationships, which refers to the perceived quality of social interactions and the support system. Higher scores in this domain could be explained by the supportive character of perceived social support [5]. Participating UYRs have diverse social contacts within the CYWS facilities with fellow resident adolescents and the professionals there. In addition, they can also build social networks outside the group homes in both educational and extracurricular contexts [44]. Despite being without their core family in a new country, their diverse social relationships may have led to higher scores in this domain compared to adult refugees, for whom it is often challenging to find new social networks in the host country [45]. However, under no circumstances should the significance of the core family’s absence be disregarded in the ongoing discussion. This is also evident in a study with Syrian adult refugees in Germany, in which those who were in Germany with their spouses reported higher QoL than those who were separated from their spouses [40]. This was also confirmed by the present study, showing higher physical and social QoL in UYRs who report more frequent contact with their family at home.

The lowest scores were observed in the domain of environmental QoL, which evaluates aspects such as safety, accommodation and opportunities for recreation. These comparatively lower scores indicated a partial dissatisfaction of the participants with their living conditions. In Germany, UYRs cannot choose where and in which CYWS facility they will stay. Furthermore, their expectations regarding possibilities of work, study, and free time are frequently not fully met [46]. For example, the area may be very rural and have few leisure activities that are of interest to UYRs. Although the accommodation in CYWS facilities has been shown to be beneficial for the mental health of refugees in comparison to refugee camps [47], the present findings show that there is still room for improvement.

### Serial mediation analysis

#### Effect of PTEs on QoL

Similar to previous studies with UYRs [26], the participants reported a high number of PTEs. We found that a higher number of PTEs was associated with lower QoL in UYRs in the physical, psychological, and social QoL domains, but not in the environmental domain. The experience of PTEs thus appears to mainly impact the physical, psychological and social QoL of UYRs. This is in line with the findings of Dangmann et al. [19], reporting that a higher number of PTEs was associated with lower QoL of young Syrian refugees in Norway. However, the present results expand these findings and highlight the importance of differentiating the dimensions of QoL, as no significant relationship was found between PTEs and environmental QoL. Thus, environmental QoL might be influenced primarily by other aspects, such as post-migration stressors.

#### Mediating role of post-migration stressors

As expected, the importance of post-migration stressors was very much emphasized by the results of the present mediation models, since for all the model’s post-migration stressors mediated the effect of PTEs on QoL alone, while controlling for mental distress. The reduction of post-migration stressors for UYRs could, therefore, have a positive direct impact on the QoL of this vulnerable population. Especially for social and environmental QoL, post-migration stressors seemed to play a crucial role, since no association between mental distress and these dimensions of QoL has been found.

The present findings were consistent with previous studies of adult refugees [5, 8] and young refugees [11, 48], suggesting a clear dose-response relationship between post-migration stressors and QoL. However, a meta-analysis on the association between daily stressors and mental health among conflict-affected forced migrants found no mediating effect of post-migration stressors on wellbeing among conflict-affected forced migrants [49]. This difference from the results of the present study can possibly be explained by the fact that only seven studies using validated QoL measures were included in the meta-analysis, as well as by the fact that this study focuses on adult refugees and their living situation might be very different compared with UYRs [50], resulting in different experiences of post-migration stressors and possibly different indicators for QoL.

#### Mediating role of mental distress

Similarly, PTSS, depressive symptoms and symptoms of anxiety mediated the effect of PTEs on physical and psychological QoL, while controlling for post-migration stressors. This association is not unexpected, as all three forms of psychological distress exhibit both physical and psychological correlates that can negatively impact the corresponding dimensions of QoL. For instance, sleep disturbances and somatic complaints are commonly observed physical manifestations of PTSS, while psychological features include intrusive thoughts and flashbacks. Similarly, aspects of depressive symptomatology are physical symptoms such as sleep disturbances and fatigue, as well as psychological aspects including persistent low mood, sadness, and hopelessness. In the case of anxiety, characteristic symptoms include vegetative responses such as heart palpitations and sweating, along with psychological symptoms such as feelings of loss of control and catastrophic thinking [51]. Interestingly, a mediating effect between PTEs and social QoL could be demonstrated only for PTSS, but not for depressive symptoms or symptoms of anxiety. This finding is unexpected, given that both depressive symptomatology and anxiety are, according to ICD-11, characterized in part by social withdrawal and avoidance of social situations [51]. Based on the present findings, social QoL, however, appears to be substantially influenced either by the experience of post-migration stressors (in the case of PTSS and symptoms of anxiety) or directly by the experience of PTEs (in the case of depressive symptoms). No mediation effect could be identified for environmental QoL, which may be attributable to the absence of a significant association between PTEs and environmental QoL.

In summary, the present study thus confirms the negative effect of mental distress on QoL in refugees [5], particularly for the physical and psychological dimensions [52] and expands the findings of a study with adult Ethiopians in refugee camps, which demonstrated a mediating role of mental distress between PTEs and QoL [20]. Interestingly, our results partially diverged from those reported by Dangmann et al. [19], where mental distress did not function as an independent mediator. A main reason for this might be that Dangmann et al. [19] did not differentiate between the four different domains in the assessment of QoL. This differentiation appears to be relevant, however, while controlling for post-migration stressors, we found different mediation effects for the different dimensions of QoL.

#### Mediating role of post-migration stressors and mental distress in series

In line with our expectations, the present study suggested the serial mediation between PTEs on physical, psychological, and partly on social QoL via post-migration stressors and mental distress, highlighting the complex relationships between these factors. QoL in UYRs might thus be influenced both by individual factors (such as experiencing PTEs before, during, and after migration and mental distress), as well as by external factors (such as experiencing discrimination or other external post-migration stressors). Thus, in line with Dangmann et al. [19], our findings support the proposal to add the domains of QoL to the ecological model of refugee distress [16] in order to generate an holistic model of well-being for young refugees.

Interestingly, the association between PTEs and social QoL partially remains even when post-migration stressors and depressive symptoms are included as mediators in the model. This suggests that experiencing PTEs might have a direct impact on social QoL. This finding may be explained by the types of PTEs that UYRs have experienced. The most frequently reported PTEs in the present study were interpersonal traumatic events, such as experiencing violence within the family or community. Given these adverse experiences with close connections, it is conceivable that UYRs have greater difficulty forming new relationships without mediating effects of post-migration stressors and depressive symptoms.

### Strengths and limitations

This investigation is one of the first to focus on QoL in UYRs in CYWS and the mediating role of both post-migration stressors and mental distress. The assessment of different dimensions of QoL allowed for a deeper understanding of the current conditions of UYRs in Germany. Moreover, the utilization of standardized self-report measures facilitated the comparison of study results with findings from previous and future studies. Additionally, the study sample was highly heterogeneous including UYRs from different countries of origin residing in different CYWS in Germany. Consequently, the current findings offer a comprehensive overview of the previously understudied topic of QoL in UYRs.

Nevertheless, several limitations to this research must be borne in mind. The results of this study may be influenced by selection bias, as participation in this study was voluntary and social workers pre-selected the UYRs they considered suitable. Second, the results are not representative for all UYRs in Germany and cannot necessarily be generalized to female UYRs because of the low number of female participants. Nevertheless, the very high proportion of male participants is typical for the group of UYRs in Germany and Europe [53]. Third, given the high pressure UYRs are under due to their asylum status, social desirability may have affected the responses of the UYRs, resulting in the underestimation of the effects. This aspect must be interpreted with caution, especially since we only used self-report data and questionnaires instead of clinical interviews to assess mental distress. Fourth, the results are only based on cross-sectional data, and do not permit any causal one-directional conclusions. Future studies should investigate these potentially bi-directional effects in longitudinal study designs in order to gain further insights into the QoL of UYRs. However, longitudinal studies are very challenging, especially in the case of UYRs, as they are a mobile group [54] who, for example, have to move out of the residential facility after reaching the age of majority. This makes it difficult to collect follow-up data. Fifth, the results have to be interpreted with caution due to the small sample size. Sixth, a reverse order of mediators is also conceivable meaning that mental distress could cause UYRs to behave and react in ways that lead them to perceive more post-migration stressors. Studies into this reverse relationship are scare and longitudinal analyses are needed to evaluate this relationship. Seventh, other refugee-specific factors may influence the reported QoL of UYRs, such as social support [5], sociocultural adaptation [5] the length of stay in the host country [5] mental health prior to migration, educational background or current medical/psychological interventions [55]. They should be assessed and taken into account for analysis in further studies in order to examine possible protective factors for QoL in UYRs. Eighth, Braig et al. [7] have shown that QoL correlated negatively with perceived COVID-related stress in young refugees. Since the data collection for the current study took place during the COVID-19 pandemic, it is possible that this had a negative impact on the reported QoL among the participants. Ninth, the reported correlations between mental distress and psychological QoL has to be interpreted with caution, since an overlap between the measured symptoms are possible [56].

### Practical implications and future research

The findings of the present study provide valuable insights with direct applications in psychosocial practice and scientific research.

On an individual level, to improve the QoL of UYRs, more specific offers of UYRs are needed. For example, in addition to the dissemination of evidence-based psychological interventions for UYRs (e.g., TF-CBT [57] or “Mein Weg” [58]), further offerings such as low-threshold stress management workshops (e.g. Self-Help+ [59]) should be widely provided. This way, depending on the level of burden and necessity, an appropriate offer could be made for UYRs, providing targeted support to cope with negative experiences such as post-migration stressors, reducing mental distress, and generally improving the QoL of UYRs. On structural level, reducing post-migration stressors, for example, through safer and more stable living conditions, support during the asylum process, or anti-discrimination measures, could lead to both a reduction in mental distress and an improvement in QoL in UYRs. Therefore, on a political level, measures to reduce post-migration stressors need to be promoted by policymakers.

This study highlights the need for further studies that examine not only the overall QoL but also the different domains of QoL individually to better capture diverse qualities and respond appropriately in practice. Against this backdrop, qualitative research could provide unique insights into the expectations and desires of UYRs in the respective domains of QoL. This could lead to specific implications on an individual and structural level to enhance long-term QoL for UYRs. Similarly, different post-migration stressors might have an different impact on the dimensions of QoL [19] and should also be differentiated in further studies.

## Conclusion

The present study has demonstrated the importance of assessing QoL in studies with UYRs. Our study demonstrated a moderate QoL in all domains, with the highest scores observed in the domain of social relationships and the lowest scores in environmental QoL. Additionally, the association of PTEs and post-migration stressors also varied depending on the type of mental distress and the respective domain of QoL. The study highlights possible improvements in policy and practice, which could have lasting positive effects on the overall well-being of UYRs.

## Electronic supplementary material

Below is the link to the electronic supplementary material.

Supplementary Material 1

## Data Availability

The datasets generated and/or analysed during the current study are not publicly available due to privacy reasons of the participants but are available from the corresponding author on reasonable request.

## Abbreviations

CATS-2: Child and Adolescents Trauma Screen
CYWS: Child and youth welfare
DSSYR: Daily Stressors Scale for Young Refugees
GAD-7: General Anxiety Disorder Scale
PHQ-9: Patient Health Questionnaire
PTE: Potentially traumatic event
PTSS: Post-traumatic stress symptoms
QoL: Quality of life
UNHCR: United Nation High Commissioner for Refugees
UYRs: Unaccompanied young refugees
WHO: World Health Organization
WHOQOL-BREF: World Health Organization Quality of Life, brief version
