# Supplementary material for: Quality of life in unaccompanied young refugees: the role of traumatic events, post-migration stressors and mental distress
Source: BMC Psychiatry. 2025 May 26;25:544. doi: 10.1186/s12888-025-06975-1 (PMC12105295; doi:10.1186/s12888-025-06975-1)
Supplement: Supplementary file 1 — Supplementary Material 1 [file 12888_2025_6975_MOESM1_ESM.docx]

**Supplementary material**

**Table A**

*Country of origin of the participants*

| Country of origin | n (%) |
| --- | --- |
| Afghanistan | 54 (33.5) |
| Syria | 21 (13.3) |
| Somalia | 16 (10.1) |
| Iran | 9 (5.7) |
| Iraq | 8 (5.1) |
| Guinea | 7 (4.4) |
| Sierra Leone | 5 (3.2) |
| Pakistan | 4 (2.5) |
| Gambia | 4 (2.5) |
| Mali | 4 (2.5) |
| Eritrea | 3 (1.9) |
| Turkey | 3 (1.9) |
| Algeria | 2 (1.3) |
| Ethiopia | 2 (1.3) |
| Tunisia | 2 (1.3) |
| Albania | 1 (0.6) |
| Angola | 1 (0.6) |
| Azerbaijan | 1 (0.6) |
| Nigeria | 1 (0.6) |
| Bangladesh | 1 (0.6) |
| Benin | 1 (0.6) |
| Cote D’Ivoire | 1 (0.6) |
| Cameroon | 1 (0.6) |
| Kenya | 1 (0.6) |
| Mongolia | 1 (0.6) |
| Romania | 1 (0.6) |
| Serbia | 1 (0.6) |
| Sudan | 1 (0.6) |
| Vietnam | 1 (0.6) |
| Morocco | 1 (0.6) |
